# FUELING SCIENTIFIC INNOVATION: NIA’S ENTREPRENEURIAL DEVELOPMENT PROGRAMS FOR AGING RESEARCHERS

**DOI:** 10.1093/geroni/igae098.0487

**Published:** 2024-12-31

**Authors:** Todd Haim

**Affiliations:** Chair

## Abstract

The symposium panel will offer a comprehensive overview of National Institute on Aging (NIA) programs designed to foster entrepreneurship and facilitate the commercialization of aging-related innovations. Central to the discussion are initiatives such as NIA Startup Challenge and Accelerator which has been demonstrated to provide first-time entrepreneurs with essential training in evidence-based technology development and position them for success. Additionally, the panel will highlight the NIA Research and Entrepreneurial Development Immersion (REDI) funding opportunities, which aim to expose researchers in gerontology and related fields to entrepreneurship.

A key focus of the panel is the pivotal role of Small Business Innovation Research (SBIR) funding as a primary source of early-stage seed funding in aging research. Panelists will elucidate how SBIR funding empowers startups to reach critical value inflection points by supporting data collection and validation processes.

Moderated by NIA entrepreneurial program leadership, the panel features representatives from Challenge finalists, SBIR-funded startups, and key stakeholders in the Aging Research-related startup ecosystem. Through diverse perspectives and real-world experiences, attendees will gain valuable insights into navigating the entrepreneurial journey, leveraging NIA resources, and harnessing the power of collaboration to enable the impactful commercialization of innovations that would enhance the health and/or care of older adults.

In summary, the panel will provide a dynamic exchange of ideas, strategies, and success stories, as we illuminate the path from research innovation to market transformation in the realm of aging research. Business and Aging Interest Group Sponsored Symposium

